# Biphasic Effect of Pirfenidone on Angiogenesis

**DOI:** 10.3389/fphar.2021.804327

**Published:** 2022-01-05

**Authors:** Donghao Gan, Wenxiang Cheng, Liqing Ke, Antonia RuJia Sun, Qingyun Jia, Jianhai Chen, Zhanwang Xu, Juan Xu, Peng Zhang

**Affiliations:** ^1^ Shenzhen Institutes of Advanced Technology, Chinese Academy of Sciences, Shenzhen, China; ^2^ School of Medicine, The Southern University of Science and Technology, Shenzhen, China; ^3^ Second Ward of Trauma Surgery Department, Linyi People’s Hospital, Linyi, China; ^4^ Department of Orthopedics, Affiliated Hospital of Shandong University of Traditional Chinese Medicine, Jinan, China; ^5^ Department of Stomatology, SijingHospital, Shanghai, China; ^6^ Shenzhen Engineering Research Center for Medical Bioactive Materials, Shenzhen, China; ^7^ University of Chinese Academy of Sciences, Beijing, China

**Keywords:** pirfenidone, angiogenesis, biphasic response, vascular endothelial cells, signaling pathways

## Abstract

Pirfenidone (PFD), a synthetic arsenic compound, has been found to inhibit angiogenesis at high concentrations. However, the biphasic effects of different PFD concentrations on angiogenesis have not yet been elucidated, and the present study used an *in vitro* model to explore the mechanisms underlying this biphasic response. The effect of PFD on the initial angiogenesis of vascular endothelial cells was investigated through a Matrigel tube formation assay, and the impact of PFD on endothelial cell migration was evaluated through scratch and transwell migration experiments. Moreover, the expression of key migration cytokines, matrix metalloproteinase (MMP)-2 and MMP-9, was examined. Finally, the biphasic mechanism of PFD on angiogenesis was explored through cell signaling and apoptosis analyses. The results showed that 10–100 μM PFD has a significant and dose-dependent inhibitory effect on tube formation and migration, while 10 nM–1 μM PFD significantly promoted tube formation and migration, with 100 nM PFD having the strongest effect. Additionally, we found that a high concentration of PFD could significantly inhibit MMP-2 and MMP-9 expression, while low concentrations of PFD significantly promoted their expression. Finally, we found that high concentrations of PFD inhibited EA.hy926 cell tube formation by promoting apoptosis, while low concentrations of PFD promoted tube formation by increasing MMP-2 and MMP-9 protein expression predominantly via the EGFR/p-p38 pathway. Overall, PFD elicits a biphasic effect on angiogenesis through different mechanisms, could be used as a new potential drug for the treatment of vascular diseases.

## Introduction

Blood vessels, including arteries, veins, and capillaries, are specifically distributed throughout various tissues and participate in different physiological processes (Kalucka et al., 2020). Abnormal angiogenesis is closely related to numerous diseases (Carulli et al., 2013); necrosis of the femoral head, osteoporosis, and other diseases are often accompanied by a decrease in angiogenesis, while vascular hyperplasia aggravates pathological processes such as rheumatoid arthritis, retinal hyperplasia, and tumors (Baeriswyl and Christofori, 2009; Alsina-Sanchís et al., 2018; MacDonald et al., 2018). Angiogenesis is a complex, multi-step process that includes activation, proliferation, migration, and the formation of tubular structures (Baeriswyl and Christofori, 2009). Angiogenetic intervention may provide a new strategy for the treatment of vascular diseases (MacDonald et al., 2018). For example, antiangiogenic drugs can be used alone or in conjunction with other drugs in the treatment of arthritis, diabetic retinopathy, tumor, which can reduce drug dose and improve the therapeutic effect (Thairu et al., 2011; Kuczynski and Reynolds, 2020). Angiogenesis-promoting drugs alone or in combination with biomaterials play an important role in promoting wound healing, myocardial regeneration, bone defect repair (Veith et al., 2019; Diomede et al., 2020; Si et al., 2020).

Pirfenidone (PFD) is a versatile pyridine compound that mainly acts on the targets of transforming growth factor (TGF)-β, and is currently widely used in the clinical treatment of diseases such as pulmonary fibrosis (Maher et al., 2020). Studies have shown that PFD can increase the expression of angiogenic cytokines and vascular endothelial growth factor (VEGF)-A in bronchoalveolar lavage fluid (Ronan et al., 2018). However, some studies have shown that PFD has no significant effect on pulmonary VEGF and capillary endothelial cell proliferation (Derseh et al., 2019). Other studies have reported that PFD can inhibit angiogenesis or its markers (Liu et al., 2017; Jiang et al., 2018a). The dual effects of the full concentration gradient of PFD on angiogenesis have not been reported. At present, targeted vascular drugs have disadvantages such as large side effects, high price, limited curative effect, or drug resistance. The development of new antiangiogenic drugs or screening of vasculogenic drugs has become a research focus in the treatment of vascular diseases (Augustin and Koh, 2017; Eelen et al., 2020). As a clinical drug, PFD has fewer side effects and has the potential to develop indications for the treatment of vascular-related diseases, which can avoid the huge risk of developing new drugs.

To explore the therapeutic effects of PFD on angiogenesis and expand its clinical utility, this study aimed to investigate the biphasic effects of various concentrations of PFD on angiogenesis in an endothelial cell model.

## Materials and Methods

### Cells and Reagents

The human umbilical vein endothelial cell line EA.hy926 was donated by the research group of Professor Qin Ling of the Chinese University of Hong Kong. anti-MMP-2 (Cat. no.40994S), Anti-p-JAK2 (Cat. no.3771S), anti-p-STAT3 (Cat. no.9145S), anti-p-SRC (Cat. no.6943T), anti-p-ERK (Cat. no.4370S), anti-p-p38 (Cat. no.9216S), anti-p-FAK (Cat. no.8556T), anti-p-AKT (Cat. no.9275S), anti-STAT3 (Cat. no.9139), anti-p-SMAD2 (Cat. no.8828S) and HRP-linked secondary antibody (cat. no.#7074) were purchased from CST. CCK-8 (Cat. no.CK04-3000T) was purchased from DOJINDO. Anti-VEGF (Cat. no.ab46154) and Anti-TGF-β (Cat. no.ab217402) antibodies were obtained from Abcam. Inhibitors of STAT3 (WP1066), ERK (Selumetinib), SRC (Dasatinib), FAK (PF-562271), P38 (SB203580), AKT (MK-2206), EGFR (EGFR-IN-12), VEGFR (SU5204), PDGFR (AG1296), as well as Pirfenidone (Cat. no.S2907) were purchased from Selleckchem. Matrigel (Cat. no.354234) and Transwell (Cat. no.3422) were purchased from Corning. TGF-β (Cat. no.10804-HNAC) was Purchased from Sinobiological. anti-MMP-9 (Cat.no.10375-2-AP) and GAPDH (Cat.no.60004-1-Ig) primary Antibodies were obtained from Proteintech (Wuhan, China). Total RNA miniprep kit (Cat. no. AP-MN-MS- -RNA-250) was Purchased from Axygen. PrimeScript™ RT Master Mix (Cat. no.RR036A) and TB Green Premix Ex Taq II (Cat. no.RR820B) were purchased from Takara.

### Cell Culture

EA. hy926 cells were cultured in H-DMEM medium (Hyclone, United States) with 100 IU/ml of penicillin/streptomycin (Sigma-Aldrich, United States) and 10% heat-inactivated fetal bovine serum (FBS; Gibco, United States) in an incubator with 5% CO_2_ at 37°C.

### Cell Viability Assay

3,000 cells/well of EA.hy926 cells were seeded into 96-well plates for 24 h and subsequently exposed to different concentrations of PFD. After 3 days of incubation (37°C, 5% CO_2_), the CCK-8 reagent was added to each well for a brief incubation period. Then, the absorbance was measured at 450 nm with a microplate reader, and the data were processed to obtain the maximum tolerated dose of PFD.

### Tube Formation Assay

The *in vitro* angiogenesis assay was established according to a previous protocol (Arnaoutova and Kleinman, 2010).

Matrigel was distributed evenly into 24-well plates and incubated at 37°C, with 5% CO_2,_ for 30 min. Then, the cell suspension and different concentrations of PFD were added to each well. After incubation for 6 h, cells were observed under a fluorescence microscope and images were taken using ImageJ medical imaging software (NIH, United States) to assess tube formation.

### Scratch Assay

When EA.hy926 cells reached 90% confluence and were in an exponential growth phase, a 200 μl pipette tip was used to make vertical scratches through the cells. After washing, a serum-free medium containing PFD was added, images were captured after 24 h, and ImageJ software was used to quantitatively analyze the degree of cell migration.

### Transwell Assay

EA.hy926 cells were seeded in the upper chamber of transwell plates, which contained a serum-free medium. The lower chamber contained a medium with 2% FBS, to which varying concentrations of PFD were added. After 4 h incubation, cells in the upper chamber were wiped with a cotton swab. The upper chamber was fixed with 4% paraformaldehyde and stained with crystal violet, and the number of cells that migrated through the membrane was observed under an inverted light microscope (OLYMPUS, Japan).

### Western Blot Analysis

EA.hy926 cellular protein was extracted following exposure to various concentrations of PFD. The protein concentration was determined through the BCA method, which was followed by gel preparation, electrophoretic separation, and protein transfer to PVDF membranes. The target proteins were detected with their respective antibodies, and GAPDH was used as an internal control. The GelView 6000 Pro instrument (BLT Photo Technology) was used for band visualization, and ImageJ software was used to quantify the target band gray value.

### Quantitative Reverse-Transcriptase PCR Analysis

Cells were seeded into 12-well plates, and after 2 days of culture, different concentrations of PFD were added to each well. Following 6 h of incubation at 37°C and 5% CO_2_, the RNA was extracted from cells on ice. The concentration was determined by NanoDrop One(Thermo Scientific, US) and reverse transcribed by T100 Thermal Cycler (Bio-Rad, US), and the target gene primers (Table 1) were added for PCR by LightCycler® 96 System (Roche, Switzerland) according to the manufacturer’s protocol. Target gene expression was evaluated by the △△Ct method after reverse transcription and amplification.

**TABLE 1:** Sequences of the primers.

Name	Forward	Reverse
MMP-2	CTT CCA AGT CTG GAG CGA TGT	TAC CGT CAA AGG GGT ATC CAT
MMP-9	CCT CTG GAG GTT CGA CGT GA	TAG GCT TTC TCT CGG TAC TGG AA
GAPDH	GGA GTC CAC TGG CGT CTT	AGG CTG TTG TCA TAC TTC TCA T

### Statistical Analyses

All data values were detected independently three times. Statistical analysis was performed by one-way analysis of variance using GraphPad Prism 8.0 software (GraphPad Software Inc., United States). Values are expressed as mean ± standard deviation (SD), with *p* < 0.05 considered significant.

## Results

### Biphasic Effect of PFD on Angiogenesis

**FIGURE 1:**
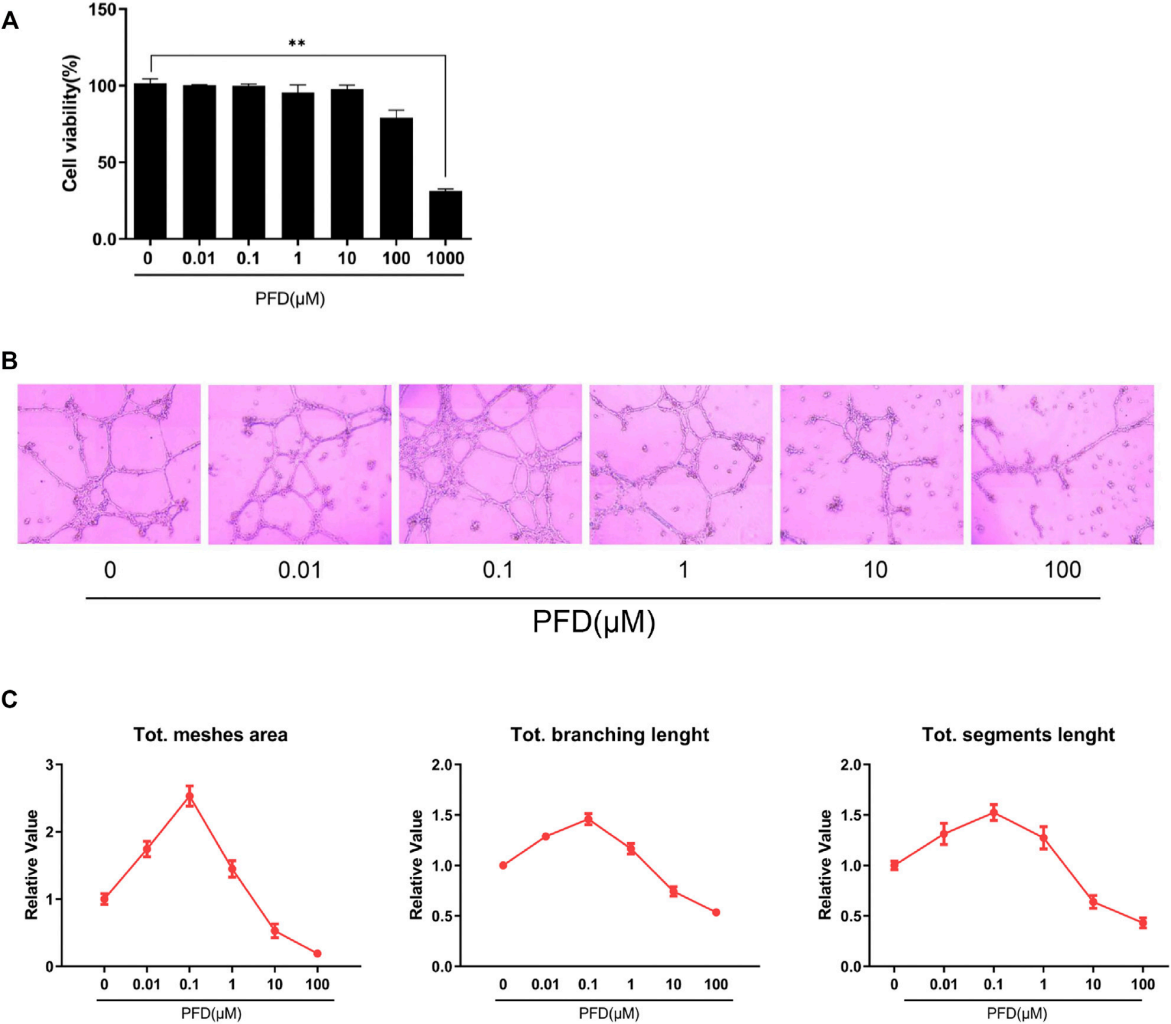
Bidirectional effects of PFD on EA.hy926 cell tube formation: **(A)** Cell viability was measured by CCK-8 assay; **(B)** The effects of different PFD concentrations on EA.hy926 cell matrigel tube formation; **(C)** Semi-quantitative analysis result of matrigel tube formation. *Compared to control cells, *p < 0.05; **p < 0.01.

To avoid the potential cytotoxic effects of PFD on EA.hy926 cells and to optimize the safe concentration range, a CCK-8 assay was used to assess cell viability. When the cells were exposed to PFD within the concentration range of 10 nM–100 μM for 72 h, there was no significant change in cell activity compared to cells in the control group, while 1 mM PFD exhibited significant toxicity (Figure 1A). Therefore, we set the maximum safe concentration of PFD to 100 μM in subsequent experiments.

Compared to cells in the control group, the inhibitory effect of PFD on the tubular formation of EA.hy926 cells was significant and concentration-dependent in the range of 10–100 μM, which could effectively reduce the mesh area, branching length, and segment length, among which the inhibitory effect of PFD at 100 μM was the most apparent (Figures 1B,C). While in the concentration range of 10 nM–1 μM, PFD significantly increased the mesh area, branching length, and segment length compared to cells in the control group. PFD at 100 nM had the most pronounced effect, however, there was a nonlinear relationship between tube formation and PFD concentration of PFD (Figures 1B,C).

**FIGURE 2:**
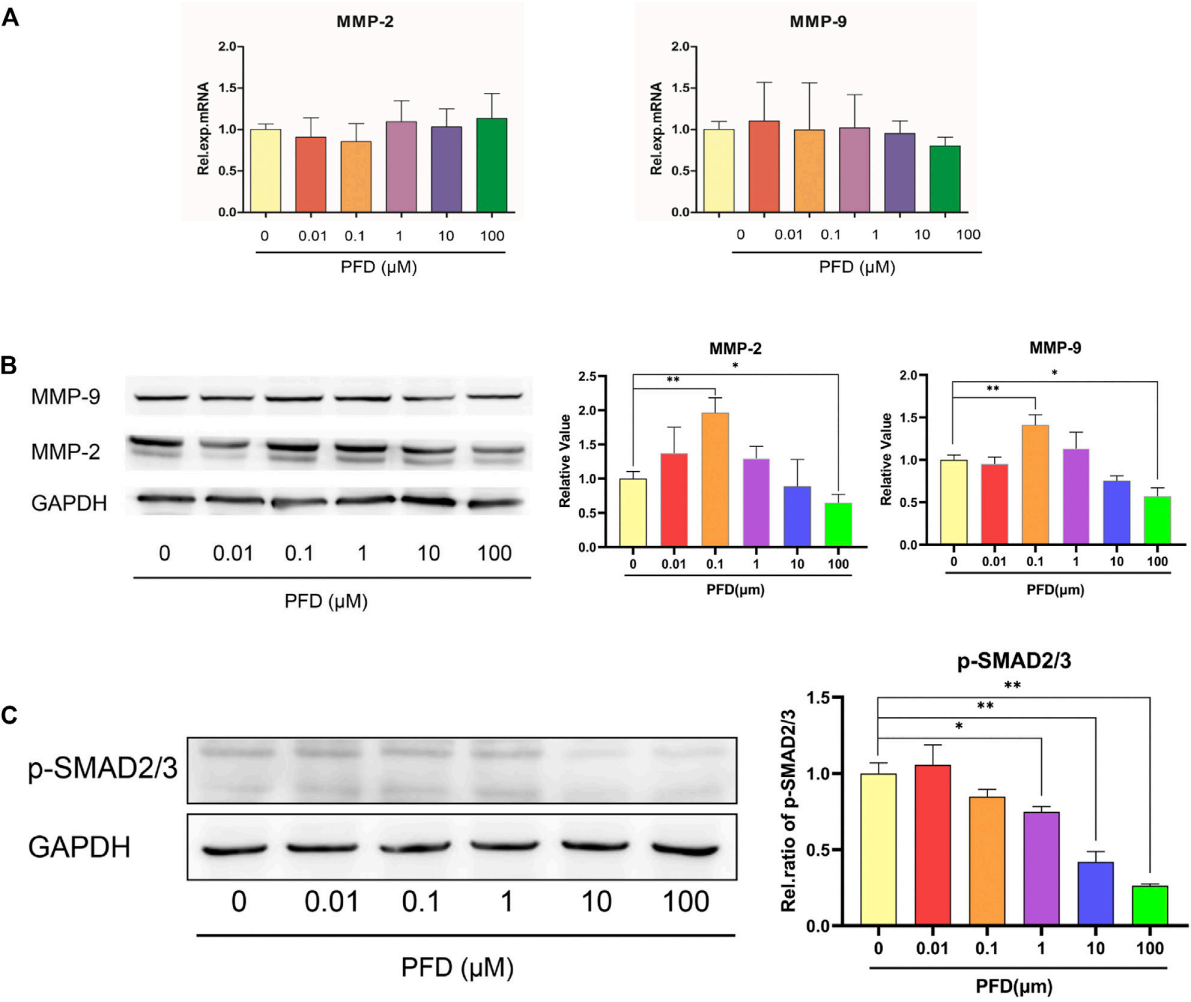
The bidirectional effect of PFD on MMPs is independent of the TGF/SMAD signaling pathway. The EA.hy926 cells were pre-treated with different concentrations of PFD for 6/24 h, and assessed by real-time qPCR and western blot analysis: **(A)** Relative mRNA expression of MMP-2 and MMP-9; **(B)** MMP-2 and MMP-9 protein expression; **(C)** p-SMAD2/3 protein expression. *p < 0.05; **p < 0.01.

The different concentrations of PFD had no significant effect on the mRNA expression of MMP-2 and MMP-9, but the effect was consistent with the tube-forming trend at the protein level (Figures 2A,B). Within the concentration range of 10–100 μM, PFD inhibited the protein expression of MMP-2 and MMP-9 in a concentration-dependent manner, among which the inhibition effect of 100 μM was the most pronounced. However, 10 nM–10 μM PFD promoted the protein expression of MMP-2 and MMP-9, of which 100 nM had the most pronounced effect. PFD significantly decreased the expression of p-SMAD2/3 in a concentration-dependent manner, indicating that PFD blocks the TGF-β/SMAD signaling pathway in a concentration-dependent manner, which was inconsistent with the trend of tube formation (Figure 2C).

### High Concentrations of PFD Inhibits EA.hy926 Cell Tube Formation by Promoting Apoptosis

**FIGURE 3:**
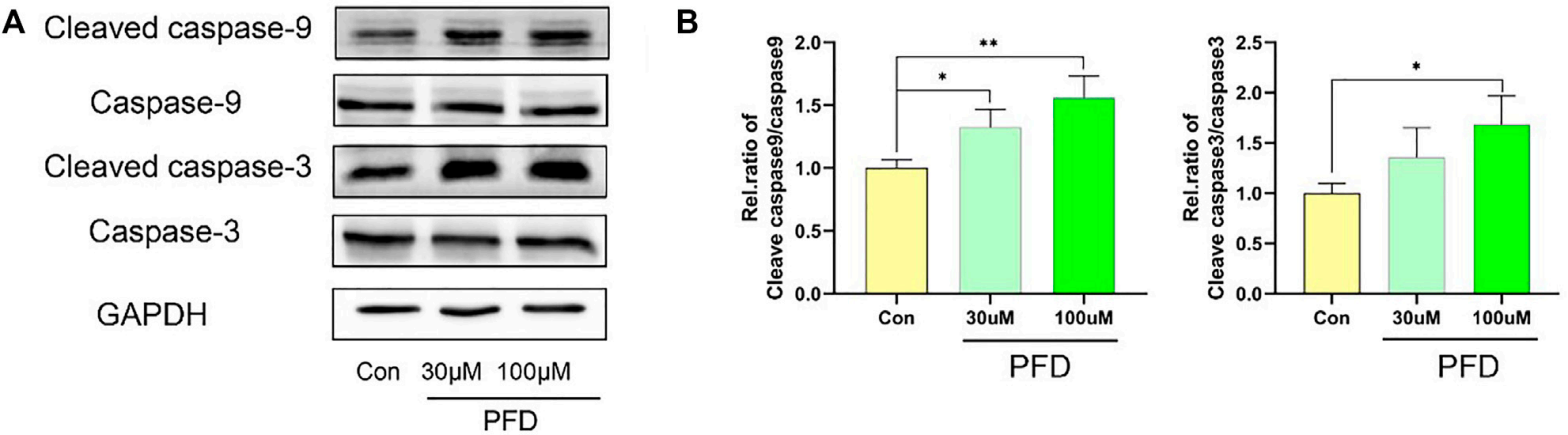
High concentrations of PFD inhibit EA.hy926 cell tube formation by promoting apoptosis. The EA.hy926 cells were stimulated with PFD (100 nM) for 24 h, followed by assessment of cleaved caspase 9, caspase 9, cleaved caspase 3, and caspase 3 expression in cell lysates by western blot analysis. **(A)** Cleaved caspase 9, caspase 9, cleaved caspase 3, and caspase 3 protein expression. **(B)** Summary of the statistical analysis, *p < 0.05; **p < 0.01.

To explore the mechanism by which PFD inhibits tube formation in EA.hy926 cells, we examined the apoptosis-related proteins cleaved caspase 9, caspase 9, cleaved caspase 3, and caspase 3, to observe the effect of PFD on apoptosis. PFD significantly increased the expression of cleaved caspase 9 and cleaved caspase 3 (Figure 3), which is consistent with the observed inhibition of tube formation and migration.

### Low Concentrations of PFD Promote Tube Formation by Increasing MMP-2 and MMP-9 Protein Expression Predominantly via the EGFR/p-p38 Pathway in EA.hy926 Cells

**FIGURE 4:**
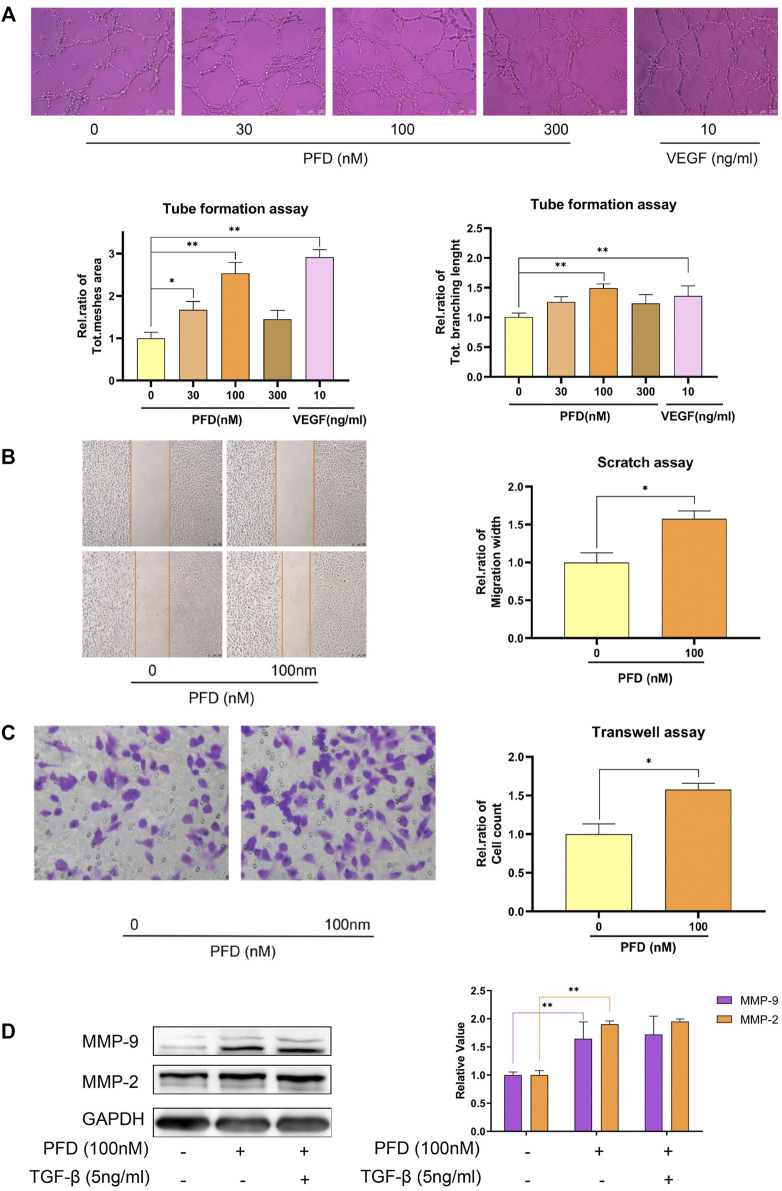
Low concentrations of PFD promote matrigel tube formation and cell migration. **(A)** Determining the optimal concentration of PFD to promote matrigel tube formation in EA.hy926 cells; **(B)** PFD promotes the recovery of the scratch width of EA.hy926 cells; **(C)** PFD promotes the transwell migration of EA.hy926 cells; **(D)** low concentrations of PFD promoted the expression of MMP-2 and MMP-9 proteins, which could not be reversed by TGF-β protein. *p < 0.05; **p < 0.01.

By further refining the concentration, we set another 3 concentrations between 10 nM and 1 μM. The results showed that PFD at 100 nM had the most obvious effect in promoting tube formation (Figure 4A). Therefore, 100 nM PFD was selected for subsequent experiments. To explore the mechanisms by which PFD promotes tube formation, we conducted a scratch test and a transwell migration experiment to show that PFD promotes angiogenesis by promoting endothelial cell migration (Figures 4B,C). To further reveal the mechanism by which PFD promotes migration, we examined the key migration cytokines, MMP-2 and MMP-9, in EA.hy926 cells after PFD stimulation. Tests showed that low concentrations of PFD promoted the expression of MMP-2 and MMP-9 proteins, which could not be reversed by TGF-β protein (Figure 4D).

**FIGURE 5:**
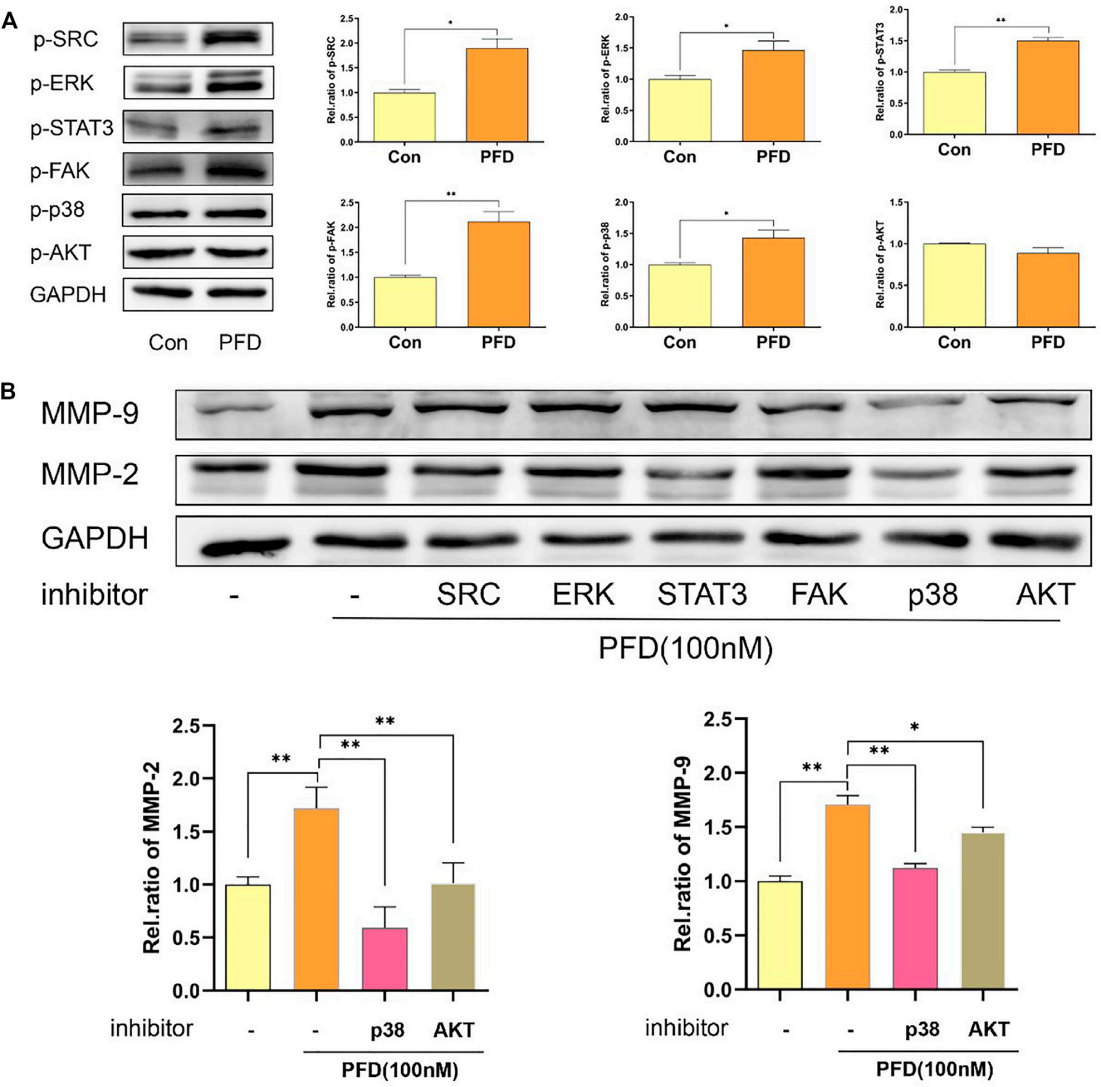
PFD promotes the expression of MMP-2 and MMP-9 predominantly via p38 pathway and is independent of the TGF-β target in EA.hy926 cells. EA.hy926 cells were pre-treated with different pharmacological inhibitors for 30 min prior to PFD (100 nM, 24 h) stimulation, which was followed by assessment of MMP-2 and MMP-9 expression in cell lysates by western blot analysis. **(A)** PFD increased phosphorylation of STAT3, SRC, p38, FAK and ERK. PFD did not have any effect on AKT phosphorylation. **(B)** Inhibitors of p38 and AKT significantly inhibited the PFD-stimulated increase in MMP-2 and MMP-9 expression. The cells were pre-treated with TGF-β (5 ng/mL) for 1 h prior to PFD (100 nM, 0.5 h) stimulation, which was followed by assessment of STAT3, SRC, AKT, p38, FAK, and ERK phosphorylation levels in cell lysates by western blot analysis. *p < 0.05; **p < 0.01. AKT-i (1 nM), SRC-i (0.1 nM), p38-i (10 µM), FAK-i (10 nM), STAT3 (0.1 µM) and ERK-i (10 nM).

To further study which signaling pathway was decisive in the PFD-induced increase in MMP-2/9 expression, we assessed the phosphorylation levels of STAT3, SRC, AKT, p38, FAK, and ERK in cell lysates by western blotting. The results showed that PFD (100 nM) significantly increased the phosphorylation of STAT3, p38, ERK, FAK, and SRC in EA.hy926 cells. PFD did not increase the phosphorylation of AKT (Figure 5A). To further support the mechanisms of these findings, we examined MMP-2 and MMP-9 protein expression after treatment with pharmacological inhibitors of STAT3, SRC, AKT, p38, FAK (10 nM), and ERK signaling pathways. The increase of MMP-2/9 expression stimulated with PFD was significantly decreased with inhibitors of AKT and p38 in EA.hy926 cells (*p* < 0.01, Figure 5B). In contrast, inhibitors of ERK, FAK, STAT3, and SRC had no obvious reversal effect on the increase in MMP-2/9 expression stimulated by PFD in EA.hy926 cells (Figure 5B). These results confirmed that PFD increases the expression of MMP-2 and MMP-9 predominantly through activation of the p38 pathway.

**FIGURE 6:**
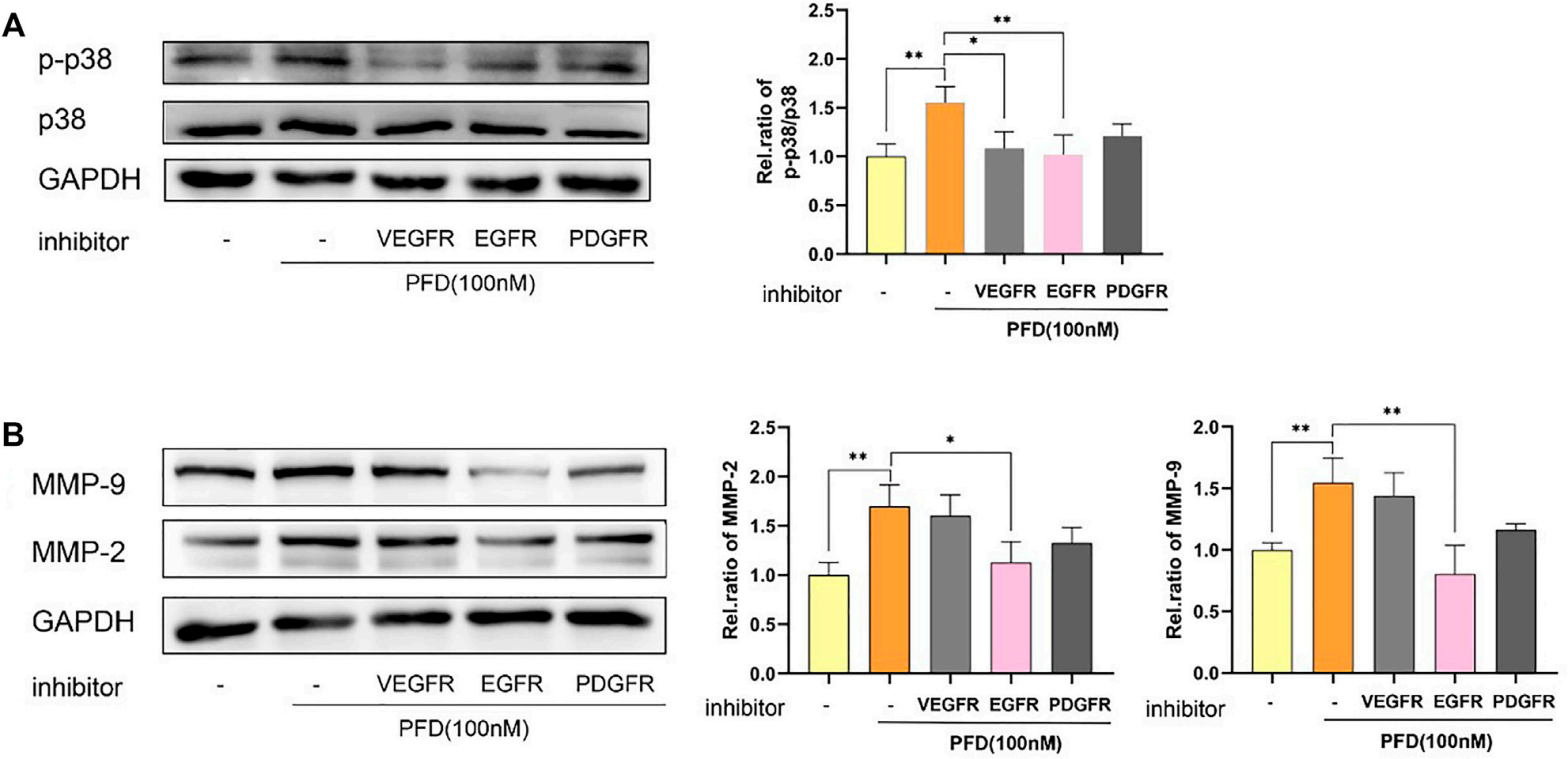
PFD increases MMP-2 and MMP-9 protein expression predominantly through activation of the p38 pathway and is EGFR-dependent. EA.hy926 cells were pre-treated with different pharmacological inhibitors for 30 min prior to PFD (100 nM, 0.5/24 h) stimulation, which was followed by assessment of p-p38 and MMP-2/9 expression in cell lysates by western blot analysis. **(A)** Inhibitors of VEGFR and EGFR significantly inhibited the PFD-stimulated increase in p-p38 expression. **(B)** Inhibitors of EGFR significantly inhibited the PFD-stimulated increasement of MMP-2 and MMP-9 expression. *p < 0.05; **p < 0.01. VEGFR-i (100 nM), EGFR (100 nM), PDGFR-i (1 µM).

Finally, we used inhibitors of Vascular Endothelial Growth Factor Receptor (VEGFR), Epidermal Growth Factor Receptor (EGFR), and Platelet-Derived Growth Factor Receptor (PDGFR) to detect whether the activation of the p38 signaling pathway by PFD was associated with angiogenesis-related VEGFR, EGFR, and PDGFR. The results showed that inhibitors of EGFR significantly inhibited the PFD-stimulated increase in p-p38 expression in EA.hy926 cells, which was consistent with the expression of MMP-2 and MMP-9 proteins (Figure 6).

## Discussion

Angiogenesis is a complex process involving the coordinated action of angiogenic factors and inhibitors, which exist in a dynamic balance under normal conditions. Vascularization plays an important role in fracture healing, bone defect repair, myocardial regeneration, wound healing, and tumor progression (Wang et al., 2015; Sivaraj and Adams, 2016; Sun et al., 2016). When the equilibrium is disrupted, that is, when the vascular system is activated (excessive angiogenesis) or inhibited (vascular degeneration), the occurrence of the disease is promoted (Yu and Wang, 2018). Under such a disequilibrium, drugs that activate or inhibit the vascular system can be used to treat diseases.

Matrix tube formation can be used to screen various factors that promote or inhibit angiogenesis and identify signal pathways related to angiogenesis regulation, which is the preferred method for evaluating angiogenesis regulatory factors (Arnaoutova and Kleinman, 2010). EA. hy926 cells can rapidly differentiate into capillary-like structures on matrigel, which can be used to simulate the initial stage of angiogenesis (Kubota et al., 1988). Previous studies have shown that high concentrations of PFD can inhibit angiogenesis or its markers (Liu et al., 2017; Jiang et al., 2018a). In this study, we confirmed that PFD has a bidirectional angiogenic effect, that is, high concentrations inhibit angiogenesis and intermediate concentrations promote blood vessel formation. First, Matrigel angiogenesis experiments showed that 10–100 µM PFD significantly and dose-dependently inhibited tube formation in EA.hy926 cells, of which 100 µM exhibited the most pronounced effect. PDF, in the concentration range of 10 nM–1 µM, significantly promoted tube formation in a non-linear manner; 100 nM had the most pronounced effect. It has been reported that the initial tube formation stage does not involve cell proliferation, but only migration (Pardali et al., 2010).

During tube formation, cells initially attach to the matrix, then migrate to each other, align and form tubes. Remodeling of the extracellular matrix is necessary for this process. *In vivo* and *in vitro* studies have shown that PFD may exert anti-fibrotic and anti-inflammatory effects by inhibiting transforming growth factor, MMP, and tumor necrosis factor expression (Waller et al., 2002; Chen et al., 2020a), and transforming growth factor and MMPs are closely related to angiogenesis (Pardali et al., 2010; Liu et al., 2018a). Studies have shown that vascular endothelial cell migration and tube-forming ability are regulated by MMPs, among which MMP-2 and MMP-9 are type IV collagen proteases, which can decompose the extracellular matrix and promote cell migration, and play an important role in the early stage of angiogenesis (Liu et al., 2018a). Up-regulation or down-regulation of MMP-2 and MMP-9 can affect vascular remodeling (Browne and Healy, 2019; Chen et al., 2020b). Here, results showed that PFD significantly changed the expression of MMP-2 and MMP-9, which is consistent with the trend of the Matrigel angiogenesis experiment, demonstrating that PFD regulates tube formation by bidirectionally regulating MMP-2 and MMP-9 in EA.hy926 cells. Through experiments, we found that the changes in MMP protein expression were not consistent with mRNA expression, indicating that PFD regulates the expression of MMP-2 and MMP-9 at the translational level. As a TGF-β inhibitor (Burghardt et al., 2007), detection showed that PFD significantly inhibited the expression of p-SMAD 2/3 and the inhibition of the TGF-β/SMAD pathway was gradually increased in the range of 10 nM–100 µM, which was inconsistent with the angiogenic curve trend. Previous studies have shown that SMAD 2/3 inhibits endothelial cell angiogenesis (Pardali et al., 2010), and inhibition of SMAD 2/3 theoretically promotes angiogenesis, which is inconsistent with this study. Therefore, it is speculated that PFD may exert angiogenic intervention effects through other pathways.

Vascular growth associated with angiogenesis must undergo a remodeling phase, including the removal of excess vascular segments and cells to form a vascular choroid system with specific physiological functions (Watson et al., 2017). Endothelial cell apoptosis plays an important role in vascular degeneration and vascular remodeling during angiogenesis, which is closely related to the process of the aortic arch, lens vascular membrane development, and wound healing, and can also disrupt the formation of the vascular network and inhibit tumor progression (Affara et al., 2007; Ehling et al., 2020; Sun et al., 2020). Studies have shown that promoting endothelial cell apoptosis can inhibit angiogenesis, which is beneficial in the treatment of diseases related to microvascular proliferation (Cao et al., 2020). Activated caspase 9 and caspase 3 are key downstream proteins in the apoptotic protein cascade and are the main indicators of apoptosis (Kang et al., 2019). We found that high concentrations of PFD can significantly enhance the expression of cleaved caspase 3 and cleaved caspase 9 proteins, activate caspase-dependent apoptosis, and reduce the expression of MMP-2 and MMP-9, thereby inhibiting tube formation.

The results showed that low concentrations of PFD promoted the expression of key migration cytokines, MMP-2 and MMP-9, and this was TGF-β target-independent. To investigate which signaling pathway was decisive in the PFD-induced increase in the expression of MMP-2/9, we evaluated AKT, ERK, p38, SRC, FAK, and STAT3 phosphorylation levels, and found that PFD may activate the p38, FAK, STAT3, ERK, and SRC pathways to promote the expression of MMP-2 and MMP-9 proteins. To support the findings obtained, we used pharmacological inhibitors of angiogenesis-related AKT, ERK, p38, SRC, FAK, and STAT3 signaling pathways (Wang et al., 2005; Karar and Maity, 2011; Zhao and Guan, 2011; Liu et al., 2018b; Batlle et al., 2019). these pathways have been reported to be associated with endothelial cell migration (Li et al., 2016; Sun et al., 2019; Zhang et al., 2019). The results of this study indicate that PFD increased MMP-2 and MMP-9 protein expression predominantly through activation of the p38 pathway, independent of the TGF-β target. Increasing studies also showed that activation of the p38 signaling pathway can up-regulate mMP2/9 expression and promote angiogenesis and tissue repair (Huang et al., 2014; Icli et al., 2019; Pan et al., 2020).

VEGFR, EGFR, and PDGFR are versatile signaling pathway integrators associated with vascular homeostasis and disease and are of great significance in wound healing and development (Andrae et al., 2008; Schreier et al., 2014; Karaman et al., 2018). Studies have shown that these molecules are down-regulated in diabetic wounds, and activation of these pathways promotes wound healing (Okonkwo et al., 2020). The molecular interaction between angiogenesis and bone formation ensures bone development, remodeling, and bone integrity. VEGF, PDGF, and EGF are the key factors in the process of angiogenesis, which can affect the coupling of angiogenesis-osteogenesis- osteoclast, and are closely related to fracture healing and bone defect repair (Yamano et al., 2014; Barabaschi et al., 2015; Unger et al., 2015).VEGFR, EGFR, and PDGFR are all receptor tyrosine kinases, (Lyle et al., 2019), and all of them can activate the p38 signaling pathway (Tsai et al., 2014; Jiang et al., 2018b; Wang et al., 2020). We found that the phosphorylation of p38 is VEGFR and EGFR-dependent, but not related to PDGFR in EA.hy926 cells stimulated by PFD.

EGFR plays a key role in endothelial cell survival, migration, and differentiation, and can regulate angiogenesis by interfering with the EGF signaling pathway (Biagioni et al., 2021). Studies have shown that the EGFR pathway may be related to wound angiogenesis, high EGFR level may lead to faster wound healing, and inhibition of EGFR may affect wound healing (Zhou et al., 2017; Simonetti et al., 2020). In addition, EGF may ameliorate drug-related osteonecrosis of the jaw by restoring vascular endothelial cell vitality and promoting angiogenesis (Wang et al., 2019). Studies have reported that the expression of MMP proteins could be activated by EGFR (Wang et al., 2018), which indicates that PFD may promote p38 phosphorylation via EGFR.

## Conclusion

**FIGURE 7:**
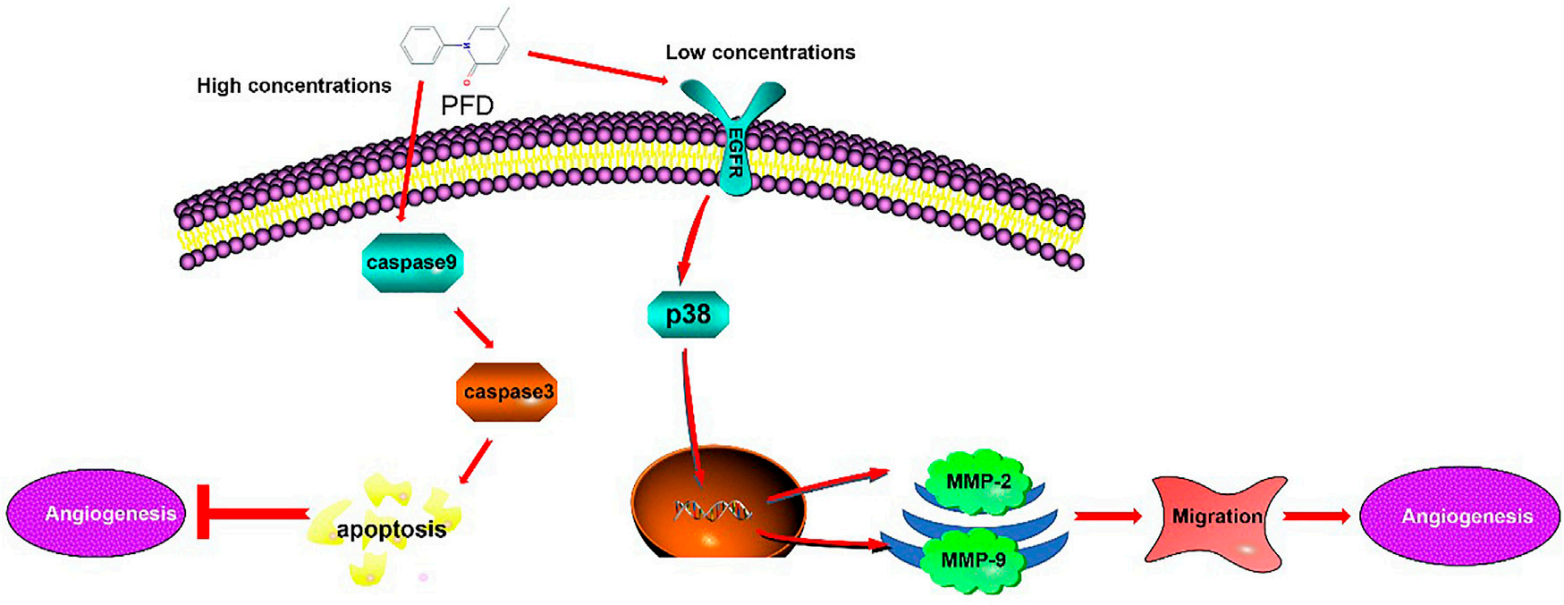
Mechanism underlying the Biphasic effect of pirfenidone on angiogenesis: PFD elicits a biphasic effect on angiogenesis through different mechanisms, whereby high concentrations inhibit angiogenesis in EA.hy926 cells by increasing apoptosis, and low concentrations promote angiogenesis through the activation of specific signaling pathways.

Our study reported, for the first time, that PFD has a bidirectional effect on angiogenesis through different mechanisms, that is, high concentrations inhibit angiogenesis through an increase in apoptosis and low concentrations promote angiogenesis through the activation of specific signaling pathways (Figure 7). These results increase the clinical utility of implementing different PFD concentrations to treat different vascular conditions. Future experiments examining the bidirectional angiogenetic effects of PFD in animal models will further elucidate its clinical potential.

## Data Availability

The raw data supporting the conclusions of this article will be made available by the authors, without undue reservation.
